# Critical Impact of Different Conserved Endoplasmic Retention Motifs and Dopamine Receptor Interacting Proteins (DRIPs) on Intracellular Localization and Trafficking of the D_2_ Dopamine Receptor (D_2_-R) Isoforms

**DOI:** 10.3390/biom10101355

**Published:** 2020-09-23

**Authors:** Kaja Blagotinšek Cokan, Maša Mavri, Catrin Sian Rutland, Sanja Glišić, Milan Senćanski, Milka Vrecl, Valentina Kubale

**Affiliations:** 1 Department of Anatomy, Histology with Embryology and Cytology, Institute of Preclinical Sciences, Veterinary Faculty, University of Ljubljana, Gerbičeva 60, 1000 Ljubljana, Slovenia; kaja.blagotinsek@mf.uni-lj.si (K.B.C.); masa.mavri@vf.uni-lj.si (M.M.); milka.vrecl@vf.uni-lj.si (M.V.); 2 School of Veterinary Medicine and Science, Medical Faculty, University of Nottingham, Sutton, Bonington Campus, Loughborough LE12 5RD, UK; Catrin.Rutland@nottingham.ac.uk; 3 Center for Multidisciplinary Research, Institute of Nuclear Sciences VINCA, University of Belgrade, Mike Petrovića Alasa 12-14, 11351 Vinča, Belgrade, Serbia; sanja@vin.bg.ac.rs (S.G.); sencanski@vin.bg.ac.rs (M.S.)

**Keywords:** D_2_ dopamine receptor, intracellular trafficking, endoplasmic reticulum, retention motifs, ICL3, interacting partners

## Abstract

The type 2 dopamine receptor D_2_ (D_2_-R), member of the G protein-coupled receptor (GPCR) superfamily, exists in two isoforms, short (D_2S_-R) and long (D_2L_-R). They differ by an additional 29 amino acids (AA) in the third cytoplasmic loop (ICL3) of the D_2L_-R. These isoforms differ in their intracellular localization and trafficking functionality, as D_2L_-R possesses a larger intracellular pool, mostly in the endoplasmic reticulum (ER). This review focuses on the evolutionarily conserved motifs in the ICL3 of the D_2_-R and proteins interacting with the ICL3 of both isoforms, specifically with the 29 AA insert. These motifs might be involved in D_2_-R exit from the ER and have an impact on cell-surface and intracellular localization and, therefore, also play a role in the function of dopamine receptor signaling, ligand binding and possible homo/heterodimerization. Our recent bioinformatic data on potential new interaction partners for the ICL3 of D_2_-Rs are also presented. Both are highly relevant, and have clinical impacts on the pathophysiology of several diseases such as Parkinson’s disease, schizophrenia, Tourette’s syndrome, Huntington’s disease, manic depression, and others, as they are connected to a variety of essential motifs and differences in communication with interaction partners.

## 1. Dopamine Receptors

G-protein-coupled receptors (GPCRs), also termed seven-transmembrane receptors (7TMRs) are by far the largest family of membrane-bound receptors, which are involved in the regulation of the neurotransmitter dopamine effects as one of their targets [1]. Based on functional, structural, and pharmacological properties, five types of dopamine receptors have been described, that belong to the D_1_- or D_2_-like subfamily of receptors (D_1_-R and D_2_-R respectively), with differing abilities of stimulation or inhibition of adenylyl cyclase (AC), respectively.

The D_1_-R subfamily is comprised of D_1_ and D_5_ receptors (D_1_-R and D_5_-R), and the D_2_ subfamily includes D_2_, D_3_ and D_4_ receptors (D_2_-R, D_3_-R, and D_4_-R). Members of the D_1_-R subfamily have a short third cytoplasmic loop (ICL3) and a very long C-terminal cytoplasmic end. In contrast, D_2_-Rs have a very long ICL3 and a short C-terminal end and include the receptor variants generated by alternative splicing (D_2_ and D_3_) or polymorphic variation (D_4_) (reviewed by Beaulieu et al.) [2]. The D_2_-R subgroup has a long ICL3 whose structure is common to the receptor interaction with the heterotrimeric protein Gα_i_ [1]. In D_1_-R, with its characteristically short ICL3, coupling with Gα_s_ proteins occurs [1,3]. The C-terminal end is approximately seven times longer in D_1_-Rs than in D_2_-Rs. Both the ICL3 and the C-terminal end are thought to serve as possible communication points for interaction with intracellular proteins. The N-terminal tail has a similar number of amino acids in all receptor subtypes and contains sites for N-glycosylation. D_1_-R and D_5_-R have two glycosylation sites, located at the N-terminal end and in extracellular loop 2 (ECL2). D_2_-R has three potential *N*-linked glycosylation sites, all in the N-terminus: N5, N17, and N23, D_3_-R has four potential glycosylation sites: N12 and N19 in the N-terminus, N97 in the first extracellular loop (ECL1), and N173 in the second extracellular loop (ECL2) [4] and D_4_-R only one in the N-terminus [5]. Cysteines located in the first and second extracellular loops (ECL1 and ECL2) are linked by a disulfide bond that stabilizes the receptor structure [6]. The endogenous ligand for the dopamine receptors is the neurotransmitter dopamine. After dopamine binds to the D_1_-R, the signaling pathway is canonically activated via the heterotrimeric protein Gα_s_ and G_olf_ G-proteins, leading to adenylate cyclase (AC) activation and cyclic adenosine monophosphate (cAMP) formation in the cell. Diversity in functional outcomes may also be achieved via selective binding to Gα_i_ and Gα_o_ proteins. Previous work has shown that D_2_-R can be stabilized by an agonist, which affect the selectivity and amount of coupling with Gα_i_ and Gα_o_ [7,8]. Although previous work had indicated that Gα_i2_ was selective for D_2L_-R [9,10], experimental data has indicated that selectivity regulation of Gα_i_ is driven by the agonist-activated conformation of D_2_-R. R(+)-3-PPP hydrochloride stimulation of D_2_-R resulted in reduced coupling with Gα_i1_ or Gα_i2_ and preferential coupling with Gα_i3_ [11]. The movement magnitude of the sixth transmembrane helix of the activated receptor was predicted to be the primary modulator of the selectivity of the G-protein subtypes [12].

Using cryo-electron microscopy, the structure of an agonist-bound activated D_2_–Gα_i_ complex reconstituted into a phospholipid membrane has been demonstrated recently [13], both as the first experimental model of a GPCR complex embedded in a phospholipid bilayer, as well as the first model of activated D_2_-R. The models revealed interactions that are unique to the membrane-embedded complex, such as conformational changes in ECL2, TM5, TM6 and TM7, propagating to the opening of the intracellular Gα_i_-binding site and helix 8 burial in the inner leaflet, ordered lysine and arginine side chains in the membrane interfacial regions, and lipid anchoring of the G-protein in the membrane [13].

Although all D-Rs recognize the same ligand, they have a differential tissue distribution and are involved in different functions in vivo [3,14]. By binding to various types of D-Rs, dopamine controls locomotor system functions, cognition, emotion, hunger, satiety, and endocrine secretion [3,5]. Impaired D_2_-R signaling is associated with the pathophysiology of many psychiatric and neurological diseases or states, including Parkinson’s disease, schizophrenia, Tourette’s syndrome, Huntington’s disease, bipolar disorder, depression, dementia, as well as others, such as restless leg syndrome and sexual dysfunction. D-Rs are an essential target for currently available modern drugs, including the dopamine precursor levodopa [3,5] for Parkinson’s disease, where dopaminergic neurons are damaged and a dopamine deficiency leads to a combination of movement and psychiatric pathologies. Thus, D-Rs are targets for motor deficits, cognitive, and motivational deficits in neuropsychiatric disorders [15]. In schizophrenia and psychosis inhibitors of D_2_-R are used to reduce increased dopaminergic signaling [16].

## 2. Dopamine Receptor Type 2 (D_2_-R)

The D_2_-R is a key component of the dopamine system that is present in two alternatively spliced transcripts of the *Drd2* gene and classified as short (D_2S_-R) and long (D_2L_-R) receptor isoforms. The long isoform differs from the short one only by the presence of an additional 29 amino acids (AA) encoded by exon 5 in the ICL3 of the D_2L_-R [17,18,19]. The inclusion is interspersed between the AA lysine (K241) and glutamic acid (E271). D_2S_-R in mice and rats are made up of 415 AA and D_2L_-R is made up of 444 AAs. Human D_2S_-R and D_2L_-R are shorter than murine and rat equivalents by one AA, consisting of 414 and 443 AAs, respectively. The isoleucine is missing between lysine (K331) and aspartic acid (D332). This region might have an essential role in the functional differences between both D_2_-R isoforms such as interactions related to G-proteins [20,21,22], post-translation modification and cell localization [11,23]. D_2_-R isoforms also indicate different in vivo functions, whereby D_2L_-R primarily acts at postsynaptic and D_2S_-R in presynaptic dopaminergic transmissions [24,25]. Data acquired on genetically engineered D_2_-R mouse model indicates additional evidence for different roles of two isoforms in cognitive and motor functions [24], responsiveness to cocaine exposure [26], and therapeutic effects of antipsychotic drugs [27]. Furthermore, they are expressed in the same cell types with more abundant expression of the D_2L_-R isoform over D_2S_-R, but with differences in their intracellular localization. While D_2S_-R is primarily localized on the plasma membrane (PM), a substantial fraction of D_2L_-R is located intracellularly, especially in the perinuclear compartments around the Golgi apparatus (GA) [14] and endoplasmic reticulum (ER) [23].

The D_2_-R is the most commonly studied dopamine receptor subtype since the majority of antipsychotic drugs act as D_2_-R antagonists in the mesolimbic dopaminergic system [28]. As a primary target for atypical and typical antipsychotic drugs and treatment of the Parkinson’s disease, many of those agents can cause potentially life-threatening and severe side effects due to the promiscuous activities against related D_2_-Rs [29]. Precisely because of this reason, it is necessary to be familiar with the details of the dopamine receptor’s complex structure and functions.

## 3. Localization Differences Between D_2S_-R and D_2L_-R

D_2_-R isoforms localization is neither species nor tissue specific. They were found in different tissues, but in highly variable ratios [25,30,31]. D_2S_-R is predominantly localized in the PM [14,22,32], whereas an intracellular D_2L_-R reservoir has been reported in the primates brain [33] and several cell lines [14,22,23].

The primary intracellular localization of the D_2L_-R in transiently transfected HEK-293, COS-7, and HeLa is the ER [23], whereas in transfected NG108-15 cells intracellularly localized D_2L_-R is predominantly co-localized with the GA matrix protein marker GM130 [14]. At the level of confocal microscopy, higher proportions of D_2L_-R than D_2S_-R were retained intracellularly in heterologous cell lines [22,23]. This finding could be due to the retention of overexpressed or incorrectly folded tagged receptors in the ER. However, immunoelectron microscopy also revealed the predominant intracellular localization of the D_2L_-R [34] in monkey dopaminergic neurons, and that these sites are cisterns of GA and ER. Ligand-promoted recruitment of the D_2L_-R [35], D_4_-R [36] and other GPCRs, such as thrombin receptors (PAR1 and PAR2), D_1_-Rs, and opioid receptors on the PM presented additional evidence for the existence of functional, pre-existing intracellular stores (reviewed by Achour, 2008) [37].

N-terminal glycosylation of different GPCR’s has a vital role in cell surface receptor expression. Mutations of potential N-terminal glycosylation sites found for D_2_-R (N5, N17, and N23) lead to decreased surface expression for D_2_-R, showing their important role in receptor distribution [38]. Fishburn et al. performed studies regarding post-translational processing of the D_2L_-R and D_2S_-R isoforms [39]. Three post-translational states were observed in both receptor isoforms: a newly synthesized protein (35 kDa), a partially glycosylated product (45 kDa) and a fully glycosylated receptor (70 kDa) [39].

A difference in the processing of the mature receptor was observed. The initial N-glycosylation of the newly synthesized receptor protein occurred shortly after synthesis in both D_2_-R isoforms, suggesting that a rapid and efficient maturation towards partially glycosylated product occurs in D_2_-R. However, a marked difference was observed in subsequent N-linked glycosylation, where D_2L_-R showed slower production of fully glycosylated proteins in comparison to the D_2S_-R. Additionally, they showed that 20% of the D_2L_-R remains in the partially processed form and never undergoes the second stage of N-linked glycosylation.

## 4. Functional Differences between D_2S_-R and D_2L_-R

Identifying probable functional differences between D_2S_-R and D_2L_-R has been the subject of numerous studies. Depending on the site of action and the effect on D_2_-R-mediated responses, isoforms have different and likely antagonistic functions in vivo [24]. D_2S_-R is mainly a presynaptic receptor, but at the postsynaptic level, it negatively modulates D_1_-R-dependent responses. In contrast, D_2L_-R is found predominantly at postsynaptic sites where it acts synergistically with D_1_-Rs [24].

The location of the inclusion in ICL3 also led to the assumption that it may affect the specificity of the interaction with G-proteins and the sequential activation of specific effector proteins.

Several studies have shown that structural differences between isoforms can determine the specificity of interactions with G-proteins [10,40]. A recent study showed the preferential coupling of both D_2_-R isoforms with G-proteins (G_i1_ and G_i2_), due to the differences in ICL3, which affect receptor behavior [11,41]. Results obtained with the messenger gene construct controlled by the cAMP response promoter suggest constitutive, i.e., agonist-independent D_2L_-R activity [42]. Studies with D_2_-R knockout mice provided additional evidence for their diverse roles in motor and cognitive functions [43], sensitivity to cocaine [44], and therapeutic/side effects of antipsychotic agents [45].

## 5. The ER Retention Motifs in GPCRs and Both D_2_-R Isoforms

The synthesis and transfer of newly formed proteins via secretory pathways from the ER to the PM is a complex process involving different mechanisms and many additional proteins and motifs that are important in protein interaction and enable their proper formation, quality control, selective retention, and transport [46]. Mechanisms that regulate the secretory transport of GPCRs or their transfer to the cell surface are poorly elucidated [47,48]. It is known that the interconnection of the same or different GPCRs, homo- and heterodimerization, is essential in the transport of GPCRs in families A and C, but not for representatives of family B on the PM [49]. Transfer of the protein to the PM requires control of transport from the ER via the GA to the PM. This process is regulated by COPI and COPII vesicles [50]. COPII-coated transfer vesicles serve anterograde transport from the ER to GA, whilst transport between GA cisternae and retrograde transport from GA to ER takes place with COPI-coated vesicles. Improperly synthesized proteins or those having exposed sequences encoding motives for retention in the ER are transported retrogradely into the ER by COPI-coated vesicles [51]. Moreover, correctly folded proteins might be retained in the ER because they hold ER retention motifs, which prevent their export from the ER. Three types of ER retention motifs have been identified in the intracellular domains of various proteins: KDEL, KKXX, and RXR type motifs [46]. The presence of specific conserved sequences, so-called ER retention signals, could be responsible for preventing D_2_-R proteins from leaving the ER. Since the level of GPCR expression dictates the magnitude of cellular responses elicited by a signal at the PM, which is the balance of elaborately regulated endocytic and exocytic trafficking, it is crucial to know the motifs as well as the proteins involved in this interplay.

## 6. KDEL and KKXX Motifs

The KDEL motif is a short C-terminal retrieval signal (Lys-Asp-Glu-Leu) identified in ER luminal chaperone proteins, such as immunoglobulin heavy chain-binding protein (BiP) and other soluble ER resident proteins [20,52,53]. The KDEL receptor recognizes this motif in the post-ER compartments, which mediates retrograde transport to the ER by COPI coatomer structures [54]. For the proper sorting of cargo into COPI vesicles, a Ras-like small GTPase ADP-ribosylation factor 1 (ARF1) activation is required [55]. ARFGAP1 activates ARF1 by hydrolysis of GTP to GDP [56]. Ligand binding on the luminal side of the KDEL receptor induces interaction with ARFGTP1 on the cytoplasmic side of the receptor, resulting in the recruitment of ARFGTP1 from the cytosol to the PM leading to ARF1 activation [57].

In zebrafish D_2_-Rs and D_3_-Rs, the expression of gene *Hsp47* was identified, which is an ER-resident collagen-specific chaperone with a C-terminal KDEL retention motif and plays a fundamental role in the folding, stability, and intracellular transport of procollagen triple helices [58].

The KDEL receptor cycles between the ER and the GA and its affinity for KDEL containing proteins changes between these two compartments. Retrieval of proteins mediated by the KDEL receptor can occur from different sites, ranging from early Golgi complex locations to trans Golgi networks [30]. In the GA, the KDEL receptor could associate with Gα_o_, one of the abundant Gα subunit and regulate receptor trafficking through G-proteins [59].

In addition to KDEL, the di-lysine motif (KKXX), have been identified as retrieval signal, important for recycling proteins from the GA back to the ER [60]. These signals are necessary for determining the localization of modified secretory and PM proteins in the ER [30].

Type I integral membrane proteins, ERGIC53, and p24 family proteins contain di-lysine KKXX motifs [46]. This carboxyl-terminal retrieval signal usually consists of two lysine residues on positions -3 and -4 relative to the C-terminus, followed by any amino acid [61]. The KKXX signal is evolutionarily conserved as it also appears in yeast [20]. Like KDEL, the KKXX motif also serves as a retrieval signal for the transport of proteins from GA by COPI vesicles, although it binds directly with coatomer structures and does not require a receptor [62].

## 7. RXR and RSRR Motifs

The RXR motif, and in some proteins, RSRR, has been found on different proteins where it disables the exit of proteins from the ER. Initially, they were found in ion channels and also in several GPCRs [46,63]. The first discovered of two arginine retention signals (the RXR and RSRR) are known to be located at the C-terminal end of the Kir 6.2 potassium channel (RXR signal) and a GPCR representative, the gamma-aminobutyric acid type B1 receptor (GABA_B1_; RSRR signal) [27]. The GABA_B1_ receptor contains the C-terminal RXR type ER retention motif RSRR, which prevents protein release from the ER. GABA_B1_ is functionally impaired in terms of ligand binding when expressed alone, whereas GABA_B2_ is nonfunctional in its signaling properties. Only when co-expressed with GABA_B2_, GABA_B1_ receptor releases from the ER and translocates to the cell surface [27,64]. Upon co-expression, the RSRR retention signal of GABA_B1_ is proposed to be masked by interaction of the C-terminus of both subunits due to highly stable α coil-coil interactions. Therefore, GABA_B1_ and GABA_B_ are functionally combined of distinct subunits as obligatory constitutive heterodimers [27,64,65,66].

**Figure 1:**
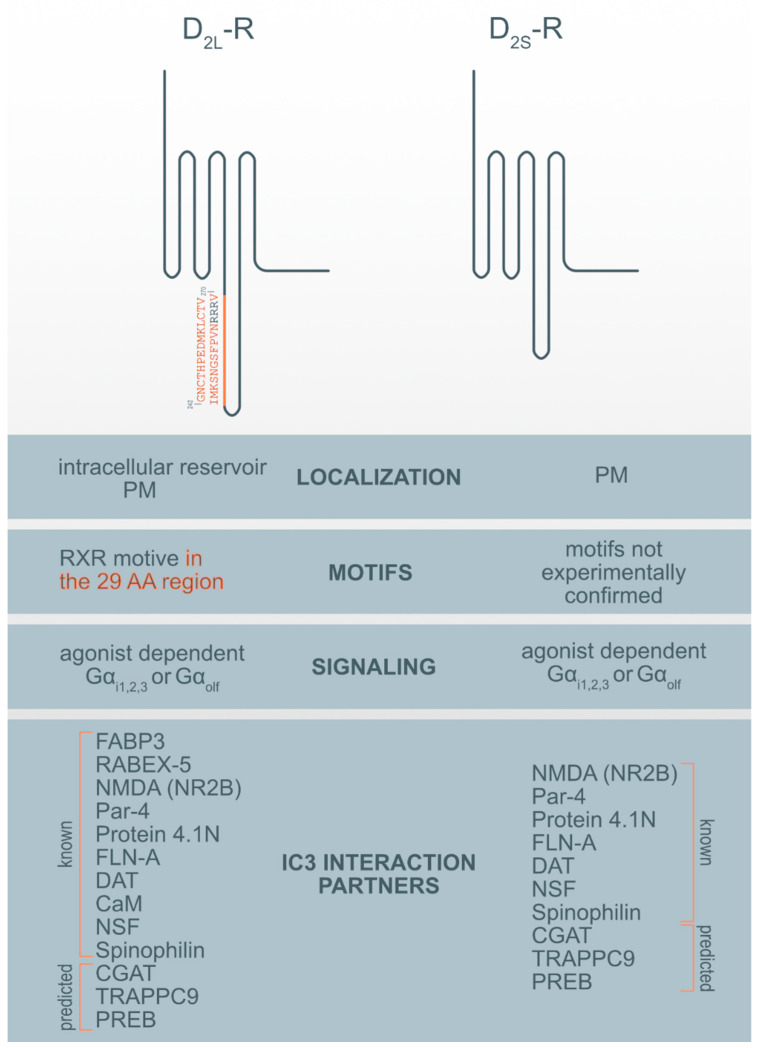
Motif and interaction partners’ differences between D_2L_-R and D_2S_-R. CaM—Ca^2+^-binding protein calmodulin; CGAT—Chromaffin granule amine transporter; DAT—dopamine transporter; FABP3-Fatty acid binding protein 3; FLN-A—filamin A; NMDA (NR2B)—NR2B subunit of the NMDA glutamate (N-methyl-D-aspartate); NSF—N-ethylmaleimide-sensitive factor; Par-4—Prostate apoptosis response-4; PM-plasma membrane; PREB—prolactin regulatory element-binding protein; Rabex-5-Rabaptin-5 interacting protein; TRAPPC9—Trafficking protein particle complex subunit 9.

There are more examples of the RXR-type retention motif in GPCR receptors. A published study showed that in the C-terminal end of the type 2c α-adrenergic receptor (α2C-AR), there is a set of five arginine residues (RRRRR), which represent a possible retention signal of the ER type RXR [67]. The RXR-type retention motif has also been described in the ICL3 of the kainate receptor, which is a ligand-dependent ion channel [68]. Disease-causing vasopressin type 2 receptors (V2R) mutations are retained in different compartments of the early secretory pathway [69]. V2R mutants connected to nephrogenic diabetes insipidus in the contrast with the wild-type V2R are less expressed on the cell surface. Additionally, the D_2_-R RXR motifs have been revealed. In our study [70], we showed that the evolutionarily conserved arginine cluster in the insert of ICL3 of D_2L_-R (R267-R269) acts as an ER retention signal and is potentially crucial for anterograde trafficking of the D_2_-R and receptor PM availability. However, we must take into account that ER exit is a highly regulated process and that one motif is not responsible solely for it. Other proposed mechanisms involving interaction with the ER-resident gatekeeper prenylated Rab acceptor 1 domain family member 3 (PRAF3) or other D_2L_-R binding and interaction proteins, such as fatty acid-binding protein 3 (FABP3), which possibly bind to 29 AA in ICL3 in D_2L_-R may be included (Figure 1) [71].

Additionally, the mutational analysis revealed that heteromers between the dopamine D_2_-R, adenosine A_2A_ and cannabinoid CB_1_ receptors are stabilized by electrostatic interactions between arginine-rich motifs in the ICL3 of D_2_-R and A_2A_ receptors and phosphorylated casein kinase 1/2 sites in ICL3 and C-tail of the CB1 receptor, and the C-terminus of the A_2A_ receptor [72]. The RXR-rich motif in ICL3 of the D_2_-R is also involved in stabilizing electrostatic interactions with a di-glutamate motif in the C-terminus of the serotonin 5-HT_2A_ and D_1_-R [73,74].

Likhite, N. et al. [75] used bioinformatics analysis to identify 583 RGG and RXR-type motifs in GPCRs. Approximately 34% of those were conserved in human GPCRs within the ICL3 and could serve as arginine methylation motifs. They showed that R217 and R219 within the ICL3 N-terminal end common to both D_2_-R isoforms serve as an arginine N-methyltransferase 5 (PRMT5) methylation motif important for modulating receptor signaling [75]. This corroborates with R217 and R219 location of within the Gα interaction domain (reviewed in [75]).

Furthermore, Table 1 summarizes selected motifs from the eukaryotic linear motif (ELM) [76] resources, which are potentially involved in ER-GA trafficking of the D_2_-R. The experimentally validated short linear motifs (SLiM) were manually curated after globular domain filtering, structural filtering, and context filtering.

## 8. ER Export Motifs in GPCRs and D_2_-R

GPCRs originate in the ER, where they are synthesized, folded, and assembled. Properly folded receptors are recruited and packaged into ER-derived COPII-coated vesicles. Transport vesicles carrying cargo receptors then migrate from the ER to the ER-Golgi intermediate complex (ERGIC), the GA, and the trans Golgi network (TGN). During their transport, receptors undergo post-translational modifications (e.g., glycosylation). Mature receptors then move from the TGN to their destination at the PM [77]. For this process, conserved sequences and motifs essential for the exit of GPCRs from the ER are critical. Export from the ER is the first step in the intracellular trafficking of GPCRs These motifs are found on the C- or N-terminal tail of the receptors and are therefore common to both D_2_-R isoforms. A triple phenylalanine motif [F(X)_3_F(X)_3_F] [14] has been identified in the membrane-proximal C-terminus of the D_1_-R that is required for receptor cell-surface expression [78]. However, no motifs have yet been identified on the D_2_-R. A newly identified ER-membrane-associated protein, DRiP78, binds to this motif, as described later.

## 9. D_2_-R Interaction Proteins (DRIPs)

More than 20 dopamine receptor-interacting membrane-associated or cytoplasmic D_2_-R interaction proteins (DRIPs) are known and several of them bind the ICL3 of the D_2_-R [79]. Using the informational spectrum method (ISM), a virtual spectroscopy method for investigating protein-protein interactions, the analysis of known interaction partners of IC3 of D_2_-R [79] was performed as previously described [80,81] and obtained the results presented in Table 2. ISM analysis of the IC3 D_2_-R interaction with protein partners corroborates with published data (reviewed in [79]) (Table 2, Figure 1). However, in addition to previously identified protein partners it has also been suggested that there are some new potential interaction partners.

Among previously described interaction partners, the highest affinity for the interaction with the D_2_-R was ascribed to *N*-methyl-D-aspartate (NMDA) receptor NR2B subunits. It was shown that a distinct region within the first 32 AA of the D_2_-R ICL3 interacts with the NR2B and disrupts the association of Ca^2+^/calmodulin-dependent protein kinase II (CaMKII) with NR2B, reduces NR2B phosphorylation at a CaMKII-sensitive site (Ser1303), and inhibits NMDA receptor-mediated currents in medium-sized striatal neurons. The D_2_-R-NR2B interaction is therefore critical for modulating NMDA receptor-mediated currents and behavioral responsiveness to cocaine [82]. The second highest propensity for interaction with the D_2_-R was observed for prostate apoptosis response-4 (Par-4). Par-4 is a protein expressed in the nervous system, where it is known to be a regulatory component in dopaminergic signaling. It is a mediator of neuronal degeneration, and is associated with the pathogenesis of Alzheimer’s disease [83]. Par-4 directly interacts with the D_2_-R via the calmodulin-binding motif in the ICL3. Furthermore, Par-4 constitutes a molecular link between impaired dopaminergic signaling and depression [84]. The N-terminal segment of the D_2_-Rs and D_3_-R was also shown to interact with neuronally enriched 4.1N protein; an interaction that contributes to the localization and stability of D_2_-Rs at the neuronal PM [85]. Similarly, filamin-A (FLN-A) also interacts with the N-terminal segment of the ICL3 of the D_2_-R and D_3_-R, and connects D-Rs with some other GPCRs, such as rhodopsin and, metabotropic glutamate receptors to the cytoskeleton, and therefore participate in their final subcellular localization [86]. The dopamine transporter (DAT) is a membrane-spanning protein that facilitate the reuptake of extracellular dopamine to the cytosol and is therefore, an essential target for cocaine, amphetamine, and some other drugs of abuse. One study showed a direct interaction between the DAT and the ICL3 (I340-Q373) of both D_2_-R isoforms. However, D_2L_-R is more capable of physically interacting with the DAT [87].

The Ca^2+^-binding protein calmodulin (CaM) binds to the N-terminal portion of the ICL3 of the D_2L_-R, within an Arg-rich epitope (VLRRRRKRVN) that is also involved in the binding to G_i/o_ proteins and the adenosine A_2A_ receptor, with the formation of A_2A_-D_2_-R heteromers [88,89]. N-ethylmaleimide-sensitive factor (NSF) is an ATPase and an essential part of the protein network responsible for different membrane fusion events, including transport through the GA and exocytosis [90]. Using immunoprecipitation and in vitro binding assays, it has been shown that NSF binds to the ICL3 of D-R (F341-Q373) and has a putative role in the interaction of D_2_-R and the Glu2 AMPA receptor [91]. Agonist stimulation of D_2_-R promotes the formation of direct protein-protein interactions between the ICL3 of the D_2_-R and the ATPase N-ethylmaleimide-sensitive factor (NSF). Spinophilin is F-actin and protein phosphatase-1-binding protein with a single PDZ domain that was identified as a protein associated with the ICL3 region of the D_2_-R. It is hypothesized to be necessary for establishing signaling complexes for dopaminergic neurotransmission through D_2_-Rs by linking receptors to downstream signaling molecules and the actin cytoskeleton [92].

Three additional hypothetical ICL3 D_2_-R interaction partners were suggested by ISM: prolactin regulatory element-binding protein (PREB), chromaffin granule amine transporter (CGAT) and trafficking protein particle complex subunit 9 (TRAPPC9). Among prospective partners, CGAT displayed the highest affinity for interacting with the ICL3 D_2_-R, followed by TRAPPC9 and PREB. For all three prospective interaction partners we were unable to find experimental evidence for the direct interaction with the ICL3 of the D_2_-R but only some indirect indication for their involvement in dopamine synthesis, transport, or D_2_-R binding. PREB is an ubiquitously expressed protein and, a member of the WD-repeat protein family, that acts as a transcriptional regulator and suppresses the expression of the adiponectin gene [93], regulates prolactin (PRL) gene expression [94] and functions as a transcriptional regulator of PRL promoter activity, and therefore might be involved in thyrotropin-releasing hormone (TRH)-induced PRL gene transcription [95]. PRL gene expression and secretion are regulated by various hormones and growth factors, including dopamine, epidermal growth factor, and thyrotropin-releasing hormone (TRH) [95]. PREB is highly expressed in the anterior pituitary. Prolactinomas are the most common pituitary tumors and are treated with the selective dopamine D_2_-R agonist cabergoline [96]. Mutation of the PREB-binding site within the promoter abrogated the ability of cabergoline to inhibit PRL promoter activity. The chromaffin granule amine transporter (CGAT), also named the vesicular monoamine transporter 1 (VMAT1), is involved in the transport of biogenic monoamines, such as serotonin, from the cytoplasm into the secretory vesicles of neuroendocrine and endocrine cells. It has a positive impact on dopamine synthesis, secretion, and transport to storage vesicles, which releases neurotransmitters into synapses as chemical messages to postsynaptic neurons [97]. The pharmaceutical industry also targets VMATs for treating hypertension, drug addiction, psychiatric disorders, Parkinson’s disease, and other neurological disorders. The trafficking protein particle complex subunit 9 (TRAPPC9), also known as NIBP, belongs to the TRAPPII multiprotein complex. TRAPPC9 is involved in vesicular trafficking from the ER to the GA and promotes the activation of NFκB signaling. It is highly expressed in the postmitotic neurons of the cerebral cortex [98].

To the best of our knowledge, only two proteins have been identified that specifically interact only with the D_2L_-R i.e., 29 AA within its ICL3. These proteins are fatty acid-binding protein 3 (FABP3) [99] and Rabaptin-5 interacting protein (Rabex-5) [23]. Fatty acid-binding protein 3 (FABP3), also named the heart-type FABP (H-FAB), is one of the novel 29 AA insert binding protein on the position (G242-V270), which also alters D_2L_-R function [99]. D_2L_-R, when activated with a ligand, is known to activate the mitogen-activated protein kinase/extracellular signal-regulated kinase (ERK) pathways, which are enhanced by FABP3 in FABP3-overexpressed cells, showing that FABP3 enhances D_2L_-R signaling [14]. A co-expression study of D_2L_-R and D_2S_-R with this protein in NG108-15 cells shows overexpression and colocalization of endogenous FABP only with the D_2L_-R in the GA and ER but not in the PM [100]. Dysfunction of FABP3 protein binding to D_2L_-R was shown in FABP3 KO mice [100], which affects emotional behavior, and is characteristic of neurodegenerative diseases such as schizophrenia and Alzheimer’s disorder. These KO mice, which showed altered sensory, motor, and emotional behaviors, also exhibited decreased methamphetamine-induced sensitization and enhanced haloperidol-induced catalepsy due to D_2_-R dysfunction. Impaired FABP brain function was observed as an essential factor in the perturbation of D_2_-R signaling [101]. Rabaptin-5 interacting protein (Rabex-5) was identified in mouse brain lysates as another protein binding the 29 AA of D_2L_-R and has been shown to promote the early-endosome formation and Rab5 activation [71]. Both proteins are essential for prolonged D_2L_-R mediated ERK signaling.

DRIPs have the propensity to bind to conserved motifs in receptors. For D_1_-R it was shown that the ER-membrane-associated protein DRiP78 binds to a FXXXFXXXF motif in the C-terminus of D_1_-R and other GPCRs. Overexpression or down-modulation of this putative two-TM domain protein leads to ER retention of D_1_-Rs, reduced ligand binding, and impaired kinetics of receptor glycosylation [48]. This mechanism acts as a chaperone and may control PM receptor targeting without traveling to the cell surface.

Some of the DRIPs are also possible “private” chaperones with other functions, escorting proteins for D_2L_-R or proteins of the quality-control machinery involved in its retention within intracellular compartments [37] and facilitating receptor cell surface expression by enabling their trafficking to the PM. Pools of intracellular D_1_-R exist in renal tubular cells, and receptor recruitment to the PM is independent of agonist activation elicited by the activation of cell surface receptors and via atrial natriuretic peptide-dependent heterologous activation [102,103].

**Table 2:** The bioinformatics approach-informational spectrum method (ISM) analysis of interaction partners of the third cytoplasmic loop (ICL3) of the D_2_-R. A lower signal to noise S/N ratio suggests a lower interaction affinity between tested protein partners.

Interaction Partner	S/N Ratio	Function	Reference
Glutamate, NMDA (NR2B)	62.39	ionotropic glutamate receptor	Liu, X.Y. et al. (2006) [82]
Par-4	48.63	regulatory component in dopamine signaling	Guo, Q. et al. (1998) [83] Park, S.K. et al. (2005) [84]
Protein 4.1N	38.61	membrane-cytoskeleton adaptor	Binda, A.V. et al. (2002) [85]
FLN-A	26.65	actin binding protein	Lin, R. et al. (2001) [86]
DAT	20.29	facilitating reuptake of extracellular dopamine back in the cytosol	Lee, F.J. et al. (2007) [87]
Gα i/z/o	17.85	binding GPCRs	
CaM	13.36	intermediate calcium-binding messenger	Navarro, G. et al. (2009) [88]
NSF	13.03	ATPase	Hanson, P.I. et. al. (1995) [90] Zou S. et al. (2005) [91]
Spinophilin	12.14	F-actin and protein phosphatase-1-binding protein	Smith, F.D. et al. (1999) [92]
**Predicted Interaction Partner**	12.14		
CGAT	19.90	involved in the transport of biogenic monoamines	
TRAPPC9	19.73	involved in vesicular trafficking from ER to GA	
PREB	18.78	transcriptional regulator	

Legend: Glutamate, NMDA (NR2B)—NR2B subunit of the NMDA glutamate receptor (N-methyl-D-aspartate); FLN-A—filamin-A; Par-4—prostate apoptosis response-4; DAT—dopamine transporter; CGAT—chromaffin granule amine transporter; TRAPPC9—trafficking protein particle complex subunit 9; PREB—prolactin regulatory element-binding protein; NSF—N-ethylmaleimide-sensitive factor; CaM—Ca^2+^-binding protein calmodulin.

## 10. Interaction with ER-Resident Gatekeeper Proteins

ER gatekeeper proteins tightly control receptor cell-surface export. GTRAP3-18, an integral ER membrane protein, was introduced as a protein both in vivo and in vitro, and is dynamically induced by retinoic acid and inhibits the activity of EAAC1 in a dose dependent manner. GTRAP3-18 forms an oligomeric complex with D_2_-R before exiting the ER, increasing the population of high-mannose oligosaccharide state proteins, and restricting its subcellular localization to the ER [104]. There is evidence that the specific gatekeeper protein PRAF2 binds to subunit GABA_1_ of the GABA_B_ receptor and prevents its progression in the biosynthetic pathway [47]. Dupre, J.D. et al. showed that one of the ER-resident proteins, which is known to regulate trafficking via a FXXXFXXXF motif of D-Rs and interact mostly with the Gγ subunit and not Gα or Gβ subunits in HEK-293 cells, is dopamine-receptor interaction protein 78 (DRiP78) [105]. Another ER-gatekeeper candidate of the D_2_-D_3_ heterodimer is an activator of G protein signaling 3 (AGS3), which binds to G_iα_GDP and inhibits GDP dissociation in the prefrontal cortex during late withdrawal from repeated cocaine administration. However, this actual mechanism in D_2_-R signaling is still unknown [106]. In familiar, as well as in sporadic Parkinson’s disease, a mutation in the leucine-reach repeat kinase 2 gene (LRRK2) represents the most frequent genetic cause of disease. LRRK2 is a member of the Roco superfamily of proteins, a novel multi-domain family of Ras-like G-proteins, involved in vesicle-mediated transport to the cell membrane. Because LRRK2 could affect D_2_-R turnover by decreasing this rate of trafficking from the GA to the CM, the LRRK2 could have an essential function in one of the possible retention mechanisms [107].

## 11. Clinical Relevance

This review examines the role of different types of conserved retention motifs and DRIPs on D_2_-R and roles in routing regulation. The impact of both is of high importance for the physiological functions of D_2L_-R and its export trafficking and precise localization in the cell. Defective transport of D_2L_-R, as well as many other GPCRs from the ER to the cell surface, is a highly regulated, dynamic process and is associated with the pathogenesis of a variety of human diseases, therefore advances in our understanding of GPCR export. Thus, the secretory pathway and its role in proper cell function are of high importance.

So far, we are not aware of any known mutation in the 29 AA region that would be associated with a disease. No variant of D_2L_-R has been linked or associated with schizophrenia, substance abuse, or alcoholism, including the most extensively investigated Ser311Cys polymorphisms of the D_2_-R gene. In vitro studies showed that the Cys311-type D_2_-R impairs dopamine-induced sequestration, which appears to be consistent with the dopamine hypothesis [108]. A naturally occurring synonymous mutation of the human D_2_-R gene (C957T, P319P) is postulated to correlate with the schizophrenia phenotype, and was shown to markedly change mRNA stability via changes in mRNA secondary structure and reduced dopamine-induced up-regulation of D_2_-R expression [109]. We also conducted a GPCR database (GPCRdb) search to find additional mutants of the D_2_-R and presented them in Table 3.

We have not found additional mutations located within the insert (AA 241–270). Further elucidation of the regulatory mechanism underlying GPCR export trafficking may provide an essential foundation for developing new therapeutic strategies in treating diseases.

The described differences in the evolutionarily preserved region within the 29 AA insertion in the ICL3 of D_2L_-R influences the regulation of D_2L_-R cellular trafficking. A thorough search through motifs showed a conserved arginine cluster within the 29-AA insert of ICL3 of the D_2L_-R, which appears to be the ER retention signal. Identifying possible candidates for DRIPs may also reveal “private” chaperones, which often display different functions or escort proteins for D_2L_-R or proteins of the quality-control machinery that play a role in GPCR retention within intracellular compartments. We speculate that other specific retention mechanisms for D_2L_-R exist. Thus, improving our knowledge of the routing regulation of these critical receptors will probably elicit the development of new therapeutic approaches in controlling the targeting of D_2L_-R at the PM.

## Figures and Tables

**Table 1:** Motifs obtained from eukaryotic linear motif (ELM) bioinformatics analysis of type 2 dopamine receptor D_2_ (D_2_-R) sequences.

ELM Name	Instances (Matched Sequence)	AA Position	ELM Description	Cell Compartment	Pattern	Probability (×10^3^)
TRG_ER_diArg_1	RRRR RRR RRKR RKRV RRR RRV	217–220 218–220 219–222 220–223 267–269 268–270	The di-Arg ER retention motif is defined by two consecutive RR residues or with a single residue insertion (RXR). The motif is completed by an adjacent hydrophobic/arginine residue, which may be on either side of the R pair.	ER membrane, integral protein, ER-GA transport vesicle membrane, ER membrane, GA-ER transport vesicle membrane, rough ER, ER	([LIVMFYWPR]R[^YFWDE]{0,1}R)|(R[^YFWDE]{0,1}R[LIVMFYWPR])	5.37
CLV_NRD_NRD_1	RRK RRV RRA RRK FRK	219–221 268–270 274–276 360–362 433–435	N-Arg dibasic convertase (NRD/Nardilysin) cleavage site (X-|-R-K or R-|-R-X).	extracellular, GA, cell surface	(.RK)|(RR[^KR])	7.47
CLV_PCSK_FUR_1	RRRK RRKRV	217–221 219–223	Furin (PACE) cleavage site (R-X-[RK]-R-|-X).	extracellular, GA, GA membrane	R.[RK]R.	50.09
CLV_PCSK_KEX2_1	KRR RRR RRR RRK KRV KRS RRR RRV RRA RRK	149–151 217–219 218–220 219–221 221–223 226–228 267–269 268–270 274–276 360–362	Yeast kexin 2 cleavage site (K-R-|-X or R-R-|-X).	extracellular, GA	[KR]R.	7.97
CLV_PCSK_PC1ET2_1	KRR KRV KRS	149–151 221–223 226–228	NEC1/NEC2 cleavage site (K-R-|-X).	extracellular, GA, GA membrane	KR.	3.90
CLV_PCSK_PC7_1	RYSSKRR RRRRKRV RVNTKRS	145–151 217–223 222–228	Proprotein convertase 7 (PC7, PCSK7) cleavage site (R-X-X-X-[RK]-R-|-X).	extracellular, GA, GA membrane	R...[KR]R.	50.09
CLV_PCSK_SKI1_1	KIAKI KKATQ RKAFL KAFLK	336–340 369–373 434–438 435–439	Subtilisin/kexin isozyme-1 (SKI1) cleavage site ([RK]-X-[hydrophobic]-[LTKF]-|-X).	ER lumen, ER GA, extracellular	[RK].[AILMFV][LTKF].	6.82
LIG_deltaCOP1_diTrp_1	EWKF	99–105	Tryptophan-based motifs enable targeting of the tethering and (dis)assembly factors to the C-terminal mu homology domain (MHD) of the coatomer subunit delta, delta-COP.	ER membrane GA membrane, COPI coated vesicle membrane, cytosol, COPI vesicle coat, transport vesicle	[DE]{1,3}.{0,2}W.{1,6}[WF]	50.10
LIG_LIR_Gen_1	EWKFSRI	99–105	Canonical LIR motif that binds to Atg8 protein family members to mediate processes involved in autophagy.	cytosol, cytoplasmic side of late endosome membrane	[EDST].{0,2}[WFY][^RKPG][^PG][ILV]	3.06
LIG_LIR_Nem_3	EWKFSRI TRYSPI	99–105 293–298	Nematode-specific variant of the canonical LIR motif that binds to Atg8 protein family members to mediate processes involved in autophagy.	cytosol, cytoplasmic side of late endosome membrane	[EDST].{0,2}[WFY]..[ILVFY]	6.36
MOD_N-GLC_1	EWKFSRI TRYSPI	99–105 293–298	Generic motif for N-glycosylation. It was shown that Trp, Asp, and Glu are uncommon before the Ser/Thr position. Efficient glycosylation usually occurs when ~60 residues or more separate the glycosylation acceptor site from the C-terminus.	extracellular, GA, ER	[EDST].{0,2}[WFY]..[ILVFY]	5.02
MOD_N-GLC_2	NEC	180–182	Atypical motif for N-glycosylation site. Examples are Human CD69, which is uniquely glycosylated at typical (Asn-X-Ser/Thr) and atypical (Asn-X-Cys) motifs, beta protein C.	extracellular, GA, ER	(N)[^P]C	29.7
TRG_ENDOCYTIC_2	YTAV YSPI	133–136 295–298	Tyrosine-based sorting signal responsible for the interaction with mu subunit of AP (Adaptor Protein) complex.	PM, clathrin-coated endocytic vesicle, cytosol	Y.[LMVIF]	2.59

Legend: GA—Golgi apparatus; ER—endoplasmic reticulum; PM—plasma membrane.

**Table 3:** A summary of mutations within the ICL3 of the D_2_-R (source: G protein-coupled receptor (GPCR) database (GPCRdb); http://gpcrdb.org/mutations/render).

Receptor	AA Residue	Location	Mutation	Reference
drd2_human	249	ICL3	D => V	Guiramand, J. et al. (1995) [110]
drd2_human	264	ICL3	P => G	Guiramand, J. et al. (1995) [110]
drd2_human	310	ICL3	P => S	Kaiser, R. et al. (2003) [111]
drd2_human	311	ICL3	S => C	Kaiser, R. et al. (2003) [111]
drd2_human	311	ICL3	S => C	Goldman D et al. (1997) [112]
drd2_mouse	251	ICL3	K => V	Guiramand, J. et al. (1995) [110]
drd2_mouse	271	ICL3	D => V	Guiramand, J. et al. (1995) [110]
drd2_rat	233	ICL3	R => G	Senogles, SE. et al. (2004) [113]
drd2_rat	234	ICL3	A => T	Senogles, SE. et al. (2004) [113]
